# Ovarian cancer risk and common variation in the sex hormone-binding globulin gene: a population-based case-control study

**DOI:** 10.1186/1471-2407-7-60

**Published:** 2007-04-05

**Authors:** Montserrat Garcia-Closas, Louise A Brinton, Jolanta Lissowska, Douglas Richesson, Mark E Sherman, Neonila Szeszenia-Dabrowska, Beata Peplonska, Robert Welch, Meredith Yeager, Witold Zatonski, Stephen J Chanock

**Affiliations:** 1 Division of Cancer Epidemiology and Genetics, National Cancer Institute, National Institutes of Health, Department of Health and Human Services, 6120 Executive Boulevard, suite 550, Rockville, MD 20952-7234, USA; 2 Core Genotyping Facility, Division of Cancer Epidemiology and Genetics, National Cancer Institute, National Institutes of Health, Department of Health and Human Services, USA; 3 Department of Cancer Epidemiology and Prevention, M. Sklodowska-Curie Institute of Oncology and Cancer Center, Warsaw, Poland; 4 Department of Occupational and Environmental Epidemiology, Nofer Institute of Occupational Medicine, Lodz, Poland

## Abstract

**Background:**

The sex hormone-binding globulin (*SHBG*) is a carrier protein that modulates the bio-availability of serum sex steroid hormones, which may be involved in ovarian cancer. We evaluated whether common genetic variation in *SHBG *and its 3' neighbor *ATP1B2*, in linkage disequilibrium, is associated with the risk of epithelial ovarian cancer.

**Methods:**

The study population included 264 women with ovarian carcinoma and 625 controls participating in a population-based case-control study in Poland. Five common single nucleotide polymorphisms (SNPs) in *SHGB *and five in *ATP1B2 *were selected to capture most common variation in this region.

**Results:**

None of the SNPs evaluated was significantly associated with ovarian cancer risk, including the putative functional SNPs *SHBG *D356N (rs6259) and -67G>A 5'UTR (rs1799941). However, our data were consistent with a decreased ovarian cancer risk associated with the variant alleles for these two SNPs, which have been previously associated with increased circulating levels of SHBG.

**Conclusion:**

These data do not support a substantial association between common genetic variation in *SHBG *and ovarian cancer risk.

## Background

Hormonal stimulation of ovarian epithelial cells has been proposed as a mechanism for carcinogenesis of the ovaries [1]. The evidence for sex steroid hormones playing a role in ovarian cancer is primarily indirect, based on animal and *in vitro *studies, as well epidemiological observations [1], while the relationship between circulating levels of sex steroids and ovarian cancer risk has not been clearly demonstrated [2,3].

The sex steroid hormone-binding globulin (*SHBG*) gene regulates the action of sex steroid hormones by modulating their bioavailability to target tissues such as the ovaries [4]. The *SHBG *gene is located on the short arm of chromosome 17. A non-synonymous SNP in exon 8 results in an amino acid substitution of asparagine for aspartic acid (D356N, rs6259) in the SHBG protein, and the asparagine (N) allele of *SHBG *has been associated with elevated circulating levels of SHBG in post-menopausal women [5-7]. An additional SNP in the 5' untranslated region (UTR) of *SHBG *has also been associated with elevated *SHBG *levels [6]. These data suggest that common variation in *SHBG *has functional relevance and thus, could affect the risk of hormonally-related cancers.

We performed a detailed assessment of common genetic variation in *SHBG *and evaluated the possibility of an association with ovarian cancer risk in the Polish Ovarian Cancer Study. We included SNPs in the *SHBG *3' flanking gene ATPase, Na+/K+ transporting beta 2 polypeptide (*ATP1B2*) because of the observation of linkage disequilibrium (LD) that extends across *SHBG *and *ATP1B2 *based on a re-sequencing study [8]. The enzyme encoded by *ATP1B2 *is involved in establishing and maintaining the electrochemical gradients of Na and K ions across the plasma membrane. Inclusion of neighboring genes in studies of relationships between disease and common genetic variation is of interest because they could affect the regulation of the protein of interest.

## Methods

### Study population

The Polish Ovarian Cancer Study is a population-based case-control study of ovarian cancer conducted among female residents of Warsaw and Lodz (Poland), 20–74 years of age. Eligible cases consisted of all women newly diagnosed with histologically confirmed ovarian carcinoma or a borderline tumor identified during the study period (June 2001 – December 2003). We identified a total of 437 eligible cases. Most (76%) cases were idenditfied through a rapid ascertainment system in participating hospitals, and the remaining cases (24%) missed by this system were identified through cancer registries. Control women were randomly selected within matching strata from complete population lists (PESEL database) of Warsaw and Lodz residents from 2001 to 2004, and were frequency matched to cases on age (5-year groups) and study site (Warsaw and Lodz). Women who reported a previous diagnosis of ovarian cancer or bilateral oopherectomy were excluded as controls. Of the eligible cases and controls, 78% and 69%, respectively, agreed to participate in the study. The main reasons for non-participation among eligible subjects included: refusal to participate (11% cases and 24% controls); unable to locate (3% cases and 7% controls), and deceased (7% cases and <0.1% controls). This resulted in 341 cases and 1994 controls participating in the main study.

All participants underwent a personal interview regarding known or suspected ovarian cancer risk factors. The median time from diagnosis to interview was 2.4 months, ranging from 5 days to one year. Of the interviewed women, 315 (92%) cases and 1911 (96%) controls provided a blood or buccal cell sample for DNA extraction; however, eight of these cases were excluded because of limited amounts of DNA or quality control problems. This report is limited to carcinomas; therefore 22 cases with borderline tumors and 21 cases with non-epithelial tumors were further excluded from the analyses, leaving 264 cases (241 with blood and 23 with buccal cell DNA). For costs-efficiency, we randomly selected a sample of 625 controls with blood DNA to approximate a 1:2 case:control ratio. Thus, final analyses included a total of 264 cases and 625 controls. All study participants were of Polish Caucasian origin. The study protocol was reviewed and approved by local and NCI Institutional Review Boards (IRBs). All participants provided written informed consent.

### Genotyping

Single nucleotide polymorphism (SNP) selection was based on a re-sequence analysis of *TP53 *and its neighboring genes, which include *SHBG *and *ATP1B2*, performed in 94 healthy Norwegian women and 102 individuals in the SNP500 Cancer panel [8]. SNPs in *SHBG *and *ATP1B2 *with a minor allele frequency >0.05 were identified during a re-sequence analysis of all exons, evolutionarily conserved regions and promoter regions and were genotyped in the Polish Ovarian Cancer Study. Genotype analyses were performed at the Core Genotyping Facility (CGF) of the Division of Cancer Epidemiology and Genetics, NCI for 5 SNPs in *SHBG *(rs1799941, -67G>A 5'UTR; rs6257, IVS1-17T>C; rs6259, Ex8+6G>A (D356N); rs727428, 1121 bp 3' of STP T>C; and rs1641544 31959 bp 3' of STP T>C); and 5 SNPs in *ATP1B2 *(rs1641536, -*8852T>C*; rs1641535 -*8703T>C*; rs1624085 x2G>C; rs1641512, *Ex7+414G>A*).

Description and methods for each genotype assay can be found at [9,10]. Duplicated DNA pairs from 70 subjects in the study showed 100% concordance for all assays. Completion was ≥ 98% for all assays. We observed no significant departures from Hardy-Weinberg equilibrium in the control population.

### Statistical Analyses

As an estimate of relative risk, odds ratios (OR) and their 95% confidence intervals (CI) were derived from unconditional logistic regression models that included dummy variables for the matching factors, i.e. age in 5 year categories and study site (Lodz or Warsaw). The association between genotypes and ovarian cancer risk was tested using a trend test, except for rare alleles in which heterozygous and homozygous variants were combined. We evaluated heterogeneity in the ORs observed for the genotype analysis on the basis of age, number of lifetime ovulatory cycles and family history of ovarian and/or breast cancer in first degree relatives, by introducing interaction terms in logistic regression models. Number of lifetime ovulatory cycles was used as a measure of a women's potential ovulatory life, and was calculated as previously described [11].

Pairwise linkage disequilibrium (LD) was estimated between SNPs based on D'and r^2 ^values using Haploview [12]. Block structure was determined using genotype data from the control population, and the solid spline of LD option (D' threshold > 0.80). Haplotype frequencies within each block, ORs and their 95%CIs were estimated using HaploStats (version 1.2.1) [13,14]. A global score statistic, adjusted for the matching factors age (in 5 year categories) and study site (Lodz or Warsaw), was used to evaluate the overall difference in haplotype frequencies between cases and controls.

## Results

The cases included in this analysis were diagnosed at a mean (SD) age of 56.2 (11.0) years. Tumor characteristics include: 45% had serous carcinomas, 62% had distal metastases, and 52% had tumors with poor grade of differentiation (Table 1). As expected, serous, mixed and unclassified tumors were frequently poorly differentiated (69%, 75% and 90%, respectively), with a lower percentage of poorly differentiated tumors among endometriod (36%) and mucinous (19%) carcinomas. Compared to control women, cases were more frequently nulliparous or had fewer full-term births, had a larger number of lifetime ovulatory cycles, and more frequently reported a first degree family history of a first-degree relative with ovarian or breast cancer (Table 1). Cases were also less likely to have used oral contraceptives. These differences were consistent with known or suspected risk factors for ovarian cancer [15].

A map of LD in the control population across the 10 SNPs evaluated is shown in Additional file 1. Three of the evaluated polymorphisms (rs1641537, rs1641536 and rs1641535 in *ATP1B1*) showed strong correlations (pairwise r^2 ^> 0.98) and, to avoid redundancy, only data for one of the correlated SNPs are shown in Table 2. Genotypes with the variant alleles for the putative functional SNPs in *SHBG*, D356N (rs6259) and -67G>A 5'UTR (rs1799941), were less common among cases than controls; however, differences were not statistically significant (Table 2). These two SNPs showed low correlation (r^2 ^= 0.03). Evaluation of joint effects suggested that subjects with genotypes carrying variant alleles for both SNPs could be at particularly low risk (OR (95%CI) for subjects with the rs6259 AG/GG and rs1799941 AG/GG genotypes (9 cases and 36 controls) was 0.47 (0.22–1.02), p = 0.06, compared to common homozygous for both SNPs (122 cases and 237 controls). However, the interaction between these two SNPs was not statistically significant (p = 0.15). None of the other SNPs evaluated were significantly associated with ovarian cancer risk. Associations with the SNPs evaluated in this report were not significantly modified by age, menopausal status, first degree family history of breast or ovarian cancer, or number of lifetime ovulatory cycles (data not shown). Associations were also not significantly modified by histological type or stage at diagnosis (data not shown).

Haplotype analyses including all non-redundant SNPs (i.e excluding rs1641536 and rs1641535 in *ATP1B1*) showed nine haplotypes with frequencies >0.01, with no significant overall association with ovarian cancer risk (global p = 0.42; Table 3). Only one haplotype, present in 5% of cases and controls, carried the D356N (rs6259) SNP and showed no significant association with risk compared to the haplotype carrying the common alleles for each of the SNPs evaluated (ATACCCCG). Although none of the individual haplotypes were significantly associated with risk, there was a borderline significant association for the most common haplotype (frequent in 35% of controls) which carried a variant for a SNP in the 5'UTR of *SHBG *(rs1799941). Data were consistent with an inverse association for women carrying this haplotype (GTACCCCG) compared to the ATACCCCG haplotype (OR 95%CI = 0.77 (0.58–1.01); Table 3).

## Discussion

This detailed evaluation of common genetic variation in *SHBG *including its neighboring gene *ATP1B2*, does not support a substantial association between common variants and ovarian cancer risk. However, the possibility of weak to modest associations could not be excluded.

The asparagine (N) allele of *SHBG *D356N and the variant allele of the -67G>A 5'UTR SNP in *SHBG *have been associated with elevated SHBG circulating levels [5-7]. Therefore, these SNP could result in a reduced bioavailability of estrogen or other sex-steroids, and a reduction in risk of hormonally-related cancers. Although the frequency of the genotypes with the variant alleles for these two SNPs was lower for cases than controls, differences were not statistically significant. A pentanucleotide (TAAAA)_n _repeat polymorphism, not measured in our study, has been found to be linked to the D356N SNP in Caucasian populations, as well as associated with elevated levels of SHBG [7]. Therefore, it is possible that functional changes not directly measured in our study might be related to risk. Given the lack of data from previous studies of ovarian cancer on the common variation in *SHBG*, further studies are needed to clarify potential relationships.

The main limitation of this study was limited power to detect weak to modest associations. Specifically, the study size provided 80% power to detect ORs of 1.5 or 0.7 for MAFs ranging from 0.10 to 0.50, assuming an additive mode of inheritance; and MAFs ranging from 0.20 to 0.35, assuming a dominant inheritance. However, the power to detect associations for MAF outside these ranges, recessive associations, or gene-gene/gene-environment interactions was low. Therefore, such associations could have missed in our study.

This population-based study has among the highest participation rate attained in population-based studies with collection of biological specimens. However, collection of DNA samples inevitably reduces overall participation rates and could introduce selection bias. This bias is unlikely to be of relevance in this report because the evaluated SNPs are unlikely to be related to potential selection factors. Further, the observed allele frequency for the *SHBG *D356N was similar to previously published studies among Caucasians [6], and the distribution of ovarian cancer risk factors in cases and controls was consistent with known or suspected risk factors for ovarian cancer [15].

## Conclusion

This report of a detailed assessment of genetic variation in *SHBG *including its neighboring gene *ATP1B2*, does not support a substantial association between common variation in this region and ovarian cancer risk. However, weak or modest associations could not be excluded, particularly a reduced risk of ovarian cancer hypothesized for the functional SNPs D356N and -67G>A 5'UTR. Therefore, further evaluation in larger study populations is warranted.

## Abbreviations

***ATP1B2 ***– ATPase, Na+/K+ transporting beta 2 polypeptide

**CI **– confidence interval

**LD **– linkage disequilibrium

**LRT **– likelihood ratio test

**MAF **– minor allele frequency

**OR **– odds ratio

***SHBG ***– sex hormone-binding globulin

**SNP **– single nucleotide polymorphisms

***TP53 ***– tumor protein p53 (Li-Fraumeni syndrome)

## Competing interests

The author(s) declare that they have no competing interests.

## Authors' contributions

MG-C participated in the design of the study and data collection, performed the statistical analysis and wrote the paper; MY, LB, RW and SJC participated in the selection and performance of genotyping assays; LAB, JL, NS-D, BP, A B-M, WZ participated in the design of the study and data collection; MES participated in the design of the study and carried out the pathology review; YQ and DR helped with the statistical analyses. All authors were involved in the drafting of the manuscript, read and approved the final manuscript.

## Pre-publication history

The pre-publication history for this paper can be accessed here:



## Supplementary Material

Additional File 1 Supplementary Figure 1: Patterns of linkage disequilibrium across the *SHBG *and its 3' neighbor gene *ATP1B2*. The figure provided shows Patterns of linkage disequilibrium across the *SHBG *and its 3' neighbor gene *ATP1B2*. Click here for file

## Figures and Tables

**Table 1:** Personal and tumor characteristics of 264 ovarian cancer cases and 625 controls in the Polish Ovarian Cancer Study.

	Cases* N = 264		Controls* N = 625		P**
Study site, N %					
Warsaw	177	67%	423	68%	0.85
Lodz	87	33%	202	32%	
Age, mean SD in years	56.3	10.8	56.1	10.8	0.83
					
No. of full-term births, N %					
Nulliparous	50	19%	79	13%	<0.0001
1	101	38%	176	28%	
2	94	36%	273	44%	
≥ 3	19	7%	97	16%	
Number of ovulatory cycles, mean SD	410.9	71.1	398.1	81.3	0.03
					
Use of oral contraceptive, N %					
Never	245	94%	556	90%	0.08
Ever	16	6%	60	10%	
					
Currently having menstrual periods, N %					
No	182	71%	401	66%	0.40
Yes	75	29%	203	34%	
Unclear	7	3%	20	3%	
					
Use of oral hormone replacement therapy among post-menopausal women					
Never	146	81%	325	82%	
Ever	35	19%	70	18%	0.15
					
Family history of breast or ovarian cancer in first-degree relatives, N %					
No	210	80%	553	88%	0.001
Breast cancer only	26	10%	24	4%	
Ovarian cancer only	26	10%	40	6%	
Both	2	1%	8	1%	
					
**Tumor Characteristics**					
Histology, N (%)					
Serous	118	45%			
Mucinous	26	10%			
Endometrioid	58	22%			
Clear cell	14	5%			
Mixed	18	7%			
Others or unclassified	30	11%			
					
Stage, N % distant	110	62%			
Not staged or missing	86				
					
Grade of differentiation, N %					
Well	21	13%			
Moderate	59	35%			
Poor	88	52%			
Missing	96				

* Differences between the total number of cases and controls and frequencies shown in the table are due to missing data.

** P values for categorical variables are based on chi squared tests, and on t-tests for continuous variables.

**Table 2:** Risk of epithelial ovarian cancer and polymorphisms in *SHBG *and its 3' neighbor *ATP1B2*, among 264 ovarian cancer cases and 625 controls in the Polish Ovarian Cancer Study*

Gene SNP		Cases	Controls	OR**	95% CI	P-trend
***SHBG***						
						
rs1799941	AA	144	278	1.00		
-67G>A	AG	118	287	0.79	0.59 – 1.07	
5'UTR	GG	23	57	0.79	0.46 – 1.33	0.14
						
rs6257	TT	242	520	1.00		
IVS1-17T>C	TC	41	91	0.97	0.65 – 1.44	
	CC	2	2	2.13	0.29 – 15.3	0.75
						
rs6259	AA	254	547	1.00		
Ex8+6G>A	AG	30	72	0.89	0.57 – 1.40	
D356N	GG	1	4	0.51	0.06 – 4.65	0.72
						
rs727428	CC	119	264	1.00		
1121 bp 3' of STP T>C	CT	121	278	0.96	0.71 – 1.30	
	TT	45	81	1.25	0.81 – 1.91	0.48
						
rs1641544	CC	261	580	1.00		
31959 bp 3' of STP T>C	CT	22	38	1.34	0.77 – 2.33	0.30
	TT	1	0			
						
***ATP1B2***						
						
rs1641537 ***	CC	215	486	1.00		
-9115T>C	CT	66	125	1.21	0.86 – 1.71	
	TT	3	8	0.90	0.23 – 3.46	0.36
						
rs1624085	CC	116	246	1.00		
Ex2G>C	CG	120	292	0.86	0.64 – 1.18	
	GG	49	84	1.25	0.82 – 1.90	0.60
						
rs1641512	GG	193	401	1.00		
Ex7+414G>A	GA	80	195	0.86	0.63 – 1.17	
	AA	12	27	0.94	0.46 – 1.90	0.42

* Differences between the total number of cases and controls and frequencies shown in the table are due to missing genotype data.

** Adjusted for age and city.

*** Highly correlated with *ATP1B1 *rs1641536 (18852T>C) and rs1641535 (-8703T>c), r^2 ^> 0.98, not shown in table to avoid redundancy.

**Table 3:** Risk of epithelial ovarian cancer and *SHBG-ATP1B2 *extended haploptypes, among 264 ovarian cancer cases and 625 controls in the Polish Ovarian Cancer Study

								Haplotype frequency		OR	95%CI	P
											
Haplotypes*								Cases	Controls			
A	T	A	C	C	C	C	G	0.26	0.20	1.00	(reference**)	
G	.	.	.	.	.	.	.	0.32	0.35	0.77	0.58 – 1.01	0.06
.	.	.	.	.	.	G	A	0.01	0.02	0.43	0.14 – 1.33	0.14
.	.	.	T	.	.	G	.	0.12	0.11	0.89	0.60 – 1.33	0.58
.	.	.	T	.	T	G	A	0.09	0.09	0.83	0.56 – 1.23	0.36
.	.	.	T	T	T	G	A	0.04	0.03	1.22	0.70 – 2.12	0.49
.	.	G	T	.	.	G	A	0.05	0.05	0.81	0.50 – 1.30	0.38
.	C	.	T	.	.	G	.	0.07	0.07	0.81	0.53 – 1.25	0.34
G	.	.	.	.	.	G	.	0.01	0.02	0.51	0.17 – 1.58	0.24
												
Global p = 0.42												

* Polymorphic bases in 5' to 3' order: *SHBG *(rs1799941, rs6257, rs6259, rs727428, rs1641544); *ATP1B2 *(rs16441537, rs1624085, rs1641512). Two *ATP1B2 *SNPs (rs1641536 and rs1641535) highly correlated (r^2 ^> 0.98) with rs1641537 are not included in the analyses.

** The reference
