# Supplementary material for: Ovarian cancer risk and common variation in the sex hormone-binding globulin gene: a population-based case-control study
Source: BMC Cancer. 2007 Apr 5;7:60. doi: 10.1186/1471-2407-7-60 (PMC1855931; doi:10.1186/1471-2407-7-60)
Supplement: Additional File 1 — Supplementary Figure 1: Patterns of linkage disequilibrium across the SHBG and its 3' neighbor gene ATP1B2. The figure provided shows Patterns of linkage disequilibrium across the SHBG and its 3' neighbor gene ATP1B2. [file 1471-2407-7-60-S1.doc]

Supplementary Figure 1: Patterns of linkage disequilibrium across the *SHBG* and its 3’ neighbor gene *ATP1B2*.
